# 晚期肺腺癌患者一线化疗后T淋巴细胞亚群变化及临床意义

**DOI:** 10.3779/j.issn.1009-3419.2012.03.06

**Published:** 2012-03-20

**Authors:** 翔 燕, 晓 赵, 顺昌 焦, 胜杰 孙, 亮亮 吴, 志勇 吴

**Affiliations:** 100853 北京，解放军总医院肿瘤内科 Department of Medical Oncology, the PLA General Hospital, Beijing 100853, China

**Keywords:** 肺肿瘤, 多西他赛, 培美曲塞, T淋巴细胞亚群, 流式细胞仪, Lung neoplasms, Docetaxol, Pemetrexed, T lymphocyte subsets, Flowcytometry

## Abstract

**背景与目的:**

机体的免疫功能异常与恶性肿瘤的发生、发展、转移及预后密切相关。T淋巴细胞亚群是反映细胞免疫功能的重要指标之一。本实验研究晚期肺腺癌患者一线化疗后外周血T淋巴细胞数量的动态变化，探讨化疗后机体免疫状态变化的动态过程，为制定化疗联合免疫治疗方案提供实验依据。

**方法:**

49例经病理学确诊的Ⅲb期-Ⅳ期肺腺癌患者，与33例正常人比较外周血T淋巴细胞数量。然后患者随机进入2个实验组，分别采用培美曲塞、顺铂联合方案及多西他赛、顺铂联合方案化疗，应用流式细胞仪检测化疗前后不同时间点淋巴细胞的组成。

**结果:**

肺癌患者的CD3^+^、CD3^+^CD4^+^、CD4^+^CD25^+^等T细胞的数量与健康对照组比较存在差异，*P*值分别为0.012，0.034和0.006；化疗后第4天及第7-10天CD3^+^、CD3^+^CD4^+^比例升高，至第21天逐渐恢复至治疗前水平；2个化疗组比较CD3^+^细胞比例均升高，而培美曲塞组第4天CD3^+^、CD3^+^CD4^+^、CD4^+^/CD8^+^升高，CD3^+^CD8^+^及CD8^+^CD28^-^比例降低，差异均具有统计学意义。部分缓解患者较早期疾病进展患者的第4天及第7-10天CD3^+^CD4^+^细胞升高；第4天CD3^+^CD8^+^、CD8^+^CD28^-^细胞降低。

**结论:**

晚期肺腺癌患者免疫功能处于抑制状态。化疗后第4天免疫功能得到一定程度恢复，至第21天恢复至治疗前水平。培美曲塞似乎对化疗后短期内免疫功能的改善有更明显的作用。化疗后免疫格局的改变可能与预后有关。

## Abstract (en)

**Background and objective:**

The immune function disorder relates closely to the occurrence, metastasis, and prognosis of cancer. T lymphocyte subsets take an important role in immune function. We identified the dynamic changes of the immune system by investigating the levels of T lymphocyte subsets in peripheral blood of lung cancer patients with advanced stage adenocarcinoma undergoing first line chemotherapy. The results aided the search for rational chemo-immunotherapy strategies in lung cancer treatment.

**Methods:**

Samples from 49 patients with pathologically demonstrated advanced stage adenocarcinoma cell lung cancer were compared with those from 33 healthy donors. Subsequently, the patients were separately treated with Docetaxol-based or Pemetrexed-based therapy. Peripheral blood samples at different time points after therapy were analyzed by flowcytometry. The lymphocyte subsets of the total lymphocytes were compared. Independent sample *t* test was used for the quantitative data analysis.

**Results:**

The percentage of CD3^+^, CD3^+^CD4^+^, CD4^+^CD25^+^ cells of the lung cancer patients significantly varied from those of the healthy donors, the P values are 0.012, 0.034 and 0.006 separately. The CD3^+^ and CD3^+^CD4^+^ levels increased significantly on the 4^th^ and 7^th^-10^th^ day post-chemotherapy, which return to normal levels on the 21^th^ day. The CD3^+^ level increased significantly both in the treatment group on all time points, while the CD3^+^, CD3^+^CD4^+^, CD4^+^/CD8^+^ levels significantly increased and the CD3^+^CD8^+^, CD8^+^CD28^-^ levels significantly decreased on the 4^th^ day in Pemetrexed group. The CD3^+^CD4^+^ levels increased significantly on the 4^th^ and 7^th^-10^th^ day and the CD3^+^CD8^+^, CD8^+^CD28^-^ levels decreased on the 4^th^ day in partial response group.

**Conclusion:**

The immune function of advanced stage adenocarcinoma cell lung cancer patients was evidently suppressed, and was restored at the 4^th^ day, followed by a reduction at the 21st day after chemotherapy. On the 4^th^ day, Pemetrexed showed better effect on the immune system. The change of immune system post-chemotherapy could be related with the prognosis.

肺癌的发病率高，预后差。晚期肺癌的治疗目前以化疗为主，但化疗的5年生存率不高，分子靶向治疗对生存率的提高也有限。肺癌患者的免疫状态与患者的肿瘤临床病理学特征以及与预后的关系成为近年来肿瘤研究焦点之一。在肿瘤细胞免疫中，T细胞起中心调控作用。T细胞分为CD4^+^、CD8^+^T细胞，根据细胞表面分子又分为几大亚类，在维持机体免疫平衡中均起着各自的作用。其中CD4^+^CD25^+^调节性T细胞亚群（Treg）具有抑制其它T细胞活化的功能^[1]^，并与肿瘤免疫逃逸有密切关系^[2]^，其数量与患者肿瘤进展程度和预后呈负相关。去除Treg或封闭其抑制功能可使肿瘤免疫恢复^[3]^。CD8^+^CD28^-^T细胞存在于肿瘤组织中，具有抑制T、B细胞活性和介导细胞毒活性两大功能。

多西他赛和培美曲塞与顺铂的联合方案为目前肺腺癌化疗最常见的一线方案。在动物实验中多西他赛已被证实可选择性抑制Treg细胞^[4]^。培美曲塞是一种多靶点抗叶酸代谢细胞毒药物，对机体免疫功能的影响未明。本研究收集64例晚期肺腺癌患者化疗前后外周静脉血，采用流式细胞技术检测其外周血中T淋巴细胞亚群占总淋巴细胞比例，与正常对照组相比较。并比较多西他赛联合顺铂与培美曲塞联合顺铂化疗对肺腺癌患者外周血中T淋巴细胞亚群的影响，以探讨化疗药物影响机体肿瘤免疫功能的机制及其可能的影响因素。

## 1 资料与方法

### 1.1 临床资料

本研究收集解放军总医院肿瘤内科2010年1月-2011年7月晚期肺腺癌患者，共64例。入选患者均为经病理确诊的肺腺癌。根据AJCC分期标准（第7版），均为Ⅲb期-Ⅳ期患者。入选前1个月内未做过其它抗肿瘤治疗，近3个月内未用过免疫增强剂，Karnofsky评分 > 70分，预计生存期 > 3个月。经解放军总医院伦理学委员会批准执行，每位入组患者签署知情同意书，随机进入多西他赛和培美曲塞治疗组。分别在治疗前、化疗第4天、化疗第7-10天（图表中为简便起见，以第10天代表）及第1周期化疗结束后1周内（第21天前后）对患者采取外周血，并进行流式细胞仪检测。15例患者因第10天或第21天数据缺失，按失访处理。最后得到并分析49例患者完整数据，年龄24岁-70岁，中位年龄51岁。其中男27例，女22例。对照组33例均为本院体检的健康者，年龄26岁-75岁，中位年龄46岁。其中男15例，女18例。

### 1.2 检测方法

用流式细胞术检测被检者外周血淋巴细胞表面分子CD3^+^、CD3^+^CD4^+^、CD3^+^CD8^+^和CD8^+^CD28^+^、CD8^+^CD28^-^、CD4^+^CD25^+^。整个操作由专人负责；流式抗体购自美国BD公司，仪器为美国BD公司的FASCalibur流式细胞仪。

T细胞亚群操作步骤为：取5 mL全血与适当体积的相应抗体及同型对照抗体混匀室温避光孵育20 min，加入2 mL流式细胞专用红细胞裂解液，避光5 min-10 min，1, 500 rpm离心5 min，弃上清，洗涤2次，加PBS 0.5 mL混匀上机，在直方图中框出淋巴细胞群，再分别计数10, 000个淋巴细胞，标记出细胞百分率。

### 1.3 统计分析

本研究所涉及数据均采用SPSS 13.0软件包进行统计分析。在T细胞亚群比例比较时，肺癌组及对照组之间采用独立样本*t*检验分析（方差不齐时，采用*t*’检验），不同时间点之间采用重复测量的方差分析（若*Mauchly's Test of Sphericity* *P* < 0.05，则以*Greenhouse-Geisser*法校正），进一步多重比较采用*LSD*法。结果以Mean±SD表示。*P* < 0.05为差异有统计学意义。

## 2 结果

### 2.1 肺癌患者组与健康组T细胞亚群的比较

肺癌患者组与健康组在性别、年龄分布无明显差异（*P* > 0.05）。表 1所示，49例肺癌患者CD3^+^、CD3^+^CD4^+^细胞比例明显低于健康组，比例分别为67.1±11.4 *vs* 72.0±6.0（*P* < 0.05），38.1±9.6 *vs* 42.0±6.4（*P* < 0.05）。CD4^+^CD25^+^（Treg）细胞比例明显高于健康组，比例为10.8±3.9 *vs* 8.4±3.5（*P* < 0.01）。CD4^+^/CD8^+^、CD8^+^CD28^+^、CD8^+^CD28^-^与健康组相比无明显差异。数据提示，肺癌患者的CD3^+^、CD3^+^CD4^+^、CD4^+^CD25^+^等T细胞的数量较健康对照组存在明显差异，而这些亚群细胞的数量和功能缺陷与肺癌患者的免疫功能状态有关（表 1）。

**1:** 肺癌组和对照组T细胞亚群检测结果（Mean±SD, %） The T lymphocyte subset of the lung cancer patient group and the control group (Mean±SD, %)

T lymphocyte subset	Lung cancer patient group (*n*=49）	Healthy group（*n*=33）	*t* (*t*’)	*P*
CD3^+^	67.1±11.4	72.0±6.0^*^	2.576^#^	0.012
CD3^+^CD4^+^	38.1±9.6	42.0±6.4^*^	2.154^#^	0.034
CD3^+^CD8^+^	25.9±7.9	26.1±6.4	0.113	0.911
CD4^+^/CD8^+^	1.5±0.8	1.8±0.7	1.431	0.156
CD4^+^CD25^+^	10.8±3.9	8.4±3.5^*^	2.843	0.006
CD8^+^CD28^+^	14.5±4.5	14.7±4.9	0.202	0.840
CD8^+^CD28^-^	15.9±7.6	15.2±6.7	0.440	0.661
^#^*t*’ values, the rests are *t* values; ^*^P＜0.05，^**^P＜0.01.				

### 2.2 肺癌患者化疗前后不同时间点T细胞亚群的变化

与化疗前比较，49例晚期肺腺癌患者化疗后每个时间点CD3^+^细胞均升高，差异有明显差异。第4天CD3^+^CD4^+^细胞比例及CD4^+^/CD8^+^升高，CD8^+^CD28^-^降低，差异具统计学意义。第7-10天CD3^+^CD4^+^细胞比例明显升高。第21天患者外周血T淋巴细胞亚群比例与化疗前基本相同，提示化疗后短期内患者免疫功能有所提高（表 2，图 1）。

**2:** 化疗前后T细胞亚群检测结果（Mean±SD, %） The T lymphocyte subset before and after chemotherapy (Mean±SD, %)

T lymphocyte subset	pro-CT^#^	The 4^th^ day of CT	The 10^th^ day of CT	The 21^th^ day of CT	*F*	*P*
CD3^+^	67.1±11.4	69.4±12.1^*^	69.8±11.2^*^	68.1±9.7^*^	21.004	< 0.001
CD3^+^CD4^+^	38.1±9.6	44.3±11.2^*^	42.3±10.3^*^	40.5±9.4	5.432	0.001
CD3^+^CD8^+^	25.9±7.9	23.1±8.3	25.0±8.5	24.7±7.4	1.250	0.294
CD4^+^/CD8^+^	1.5±0.8	2.0±1.1^*^	1.8±1.0	1.6±1.0	2.333	0.077
CD4^+^CD25^+^	10.8±3.9	11.1±3.2	10.5±3.5	10.3±3.3	0.641	0.590
CD8^+^CD28^+^	14.5±4.5	13.9±4.0	14.0±4.5	14.4±4.2	0.290	0.833
CD8^+^CD28^-^	15.9±7.6	12.1±7.9^*^	14.8±8.1	14.6±7.7	2.138	0.098
^#^CT is abbreviation of chemotherapy; ^*^P < 0.05 compared to pro-CT.						

**1:**
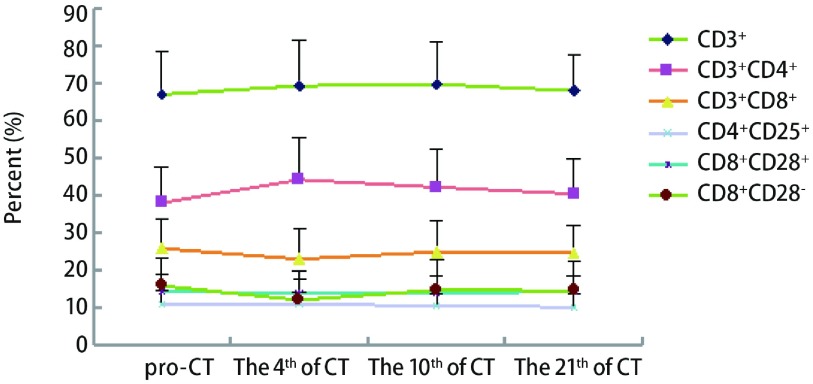
T细胞亚群化疗前后比例变化趋势图 The trend of T lymphocyte subset before and after CT (chemotherapy)

### 2.3 多西他赛组与培美曲塞组化疗前后T淋巴细胞的变化

将多西他赛+顺铂组与培美曲塞+顺铂组进行分层分析。多西他赛组共纳入23例患者，培美曲塞组共纳入26例患者。两组在性别、年龄方面比较无明显差异。同样将两组患者化疗后第4、7-10及21天的外周血T淋巴细胞进行比较，结果显示：多西他赛化疗后各个时间点CD3^+^均升高，差异有统计学意义；其它细胞亚群在化疗后未出现明显变化。而培美曲塞组化疗第4天CD3^+^、CD3^+^CD4^+^、CD4^+^/CD8^+^升高，CD3^+^CD8^+^及CD8^+^CD28^-^比例降低，差异均具有统计学意义。化疗后第7-10天CD3^+^、CD3^+^CD4^+^及CD4^+^/CD8^+^仍明显升高，差异具有统计学意义（图 2，图 3，表 3）。

**2:**
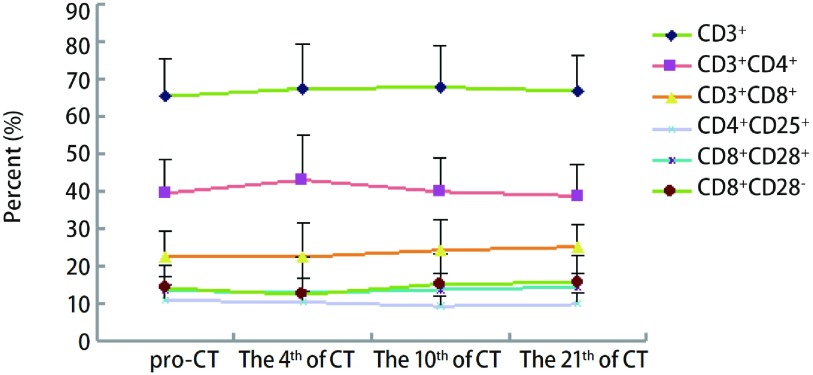
多西他赛组化疗前后T淋巴细胞变化趋势图 The trend of T lymphocyte subset in doxetaxol group

**3:**
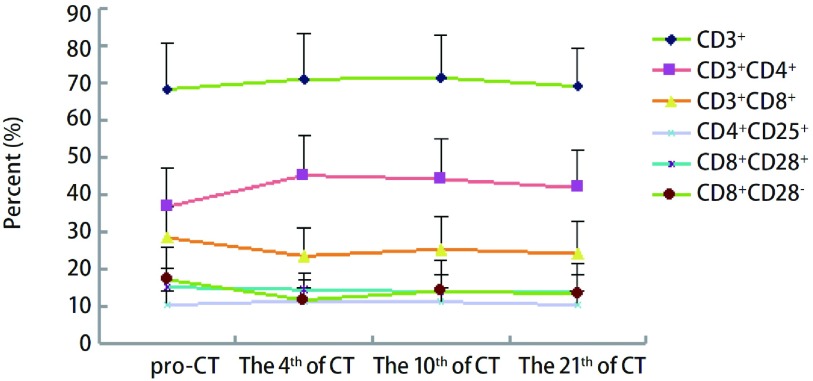
培美曲塞组化疗前后T淋巴细胞变化趋势图 The trend of T lymphocyte subset in pemetrexed group

**3:** 不同化疗药物组T细胞亚群检测结果（Mean±SD, %） The T lymphocyte subset in groups of different chemotherapy drugs(Mean±SD, %)

T lymph-ocyte subset	CT^&^ regimen	pro-CT	The 4^th^ day of CT	The 10^th^ day of CT	The 21^th^ day of CT	*F*	*P*
CD3^+^	Docetaxol	65.5±10.1	67.5±12.0^*^	67.9±10.9^*^	66.9±9.3^*^	6.381	0.002
	Pemetrexed	68.4±12.5	71.1±12.2^*^	71.5±11.3^*^	69.2±10.1	16.746	< 0.001
	Sum	67.1±11.4	69.4±12.1	69.8±11.2	68.1±9.7	20.585	< 0.001
	*t*	2.305	1.402	1.368	1.086	*F*=1.198^※^ *P*=0.313	
	*P*	0.025	0.167	0.177	0.282		
CD3^+^CD4^+^	Docetaxol	39.6±8.9	43.2±12.1	40.0±9.2	38.9±8.4	1.208	0.308
	Pemetrexed	36.9±10.2	45.3±10.5^*^	44.3±10.9^*^	42.0±10.2 ^*^	6.823	< 0.001
	Sum	38.1±9.6	44.3±11.2	42.3±10.3	40.5±9.4	5.245	0.002
	*t*	0.235	0.856	1.508	0.571	*F*=1.888^※^ *P*=0.134	
	*P*	0.815	0.396	0.138	0.570		
CD3^+^CD8^+^	Docetaxol	22.8±6.5	22.6±9.2	24.4±8.1	25.3±6.1	0.786	0.506
	Pemetrexed	28.6±8.2	23.7±7.6^*^	25.5±8.9	24.3±8.5	2.595	0.059
	Sum	25.9±7.9	23.2±8.3	25.0±8.5	24.7±7.4	1.169	0.324
	*t*	3.167	0.716	0.689	0.372^#^	*F*=2.092^※^ *P*=0.104	
	*P*	0.002	0.477	0.494	0.711		
CD4^+^/CD8^+^	Docetaxol	1.6±1.0	2.0±1.1	1.8±1.0	1.4±0.8	1.568	0.205
	Pemetrexed	1.4±0.7	2.0±1.0^*^	1.9±1.1^*^	1.8±1.1	1.735	0.167
	Sum	1.5±0.8	2.0±1.1	1.8±1.0	1.6±1.0	2.329	0.077
	*t*	0.124	0.238	0.452	1.408	*F*=0.973^※^ *P*=0.408	
	*P*	0.902	0.813	0.653	0.165		
CD4^+^CD25^+^	Docetaxol	11.0±4.1	10.6±3.0	9.5±2.8	10.0±2.9	0.871	0.460
	Pemetrexed	10.6±3.9	11.7±3.4	11.4±3.8	10.6±3.7	0.691	0.561
	Sum	10.8±3.9	11.2±3.2	10.5±3.5	10.3±3.3	0.651	0.583
	*t*	0.209	0.901	1.929	0.116	*F*=0.923^※^ *P*=0.431	
	*P*	0.835	0.372	0.059	0.908		
CD8^+^CD28^+^	Docetaxol	13.7±3.6	13.0±3.7	13.9±4.4	14.6±3.7	0.939	0.427
	Pemetrexed	15.3±5.1	14.7±4.2	14.1±4.6	14.2±4.6	0.423	0.737
	Sum	14.5±4.5	13.9±4.0	14.0±4.5	14.4±4.2	0.302	0.824
	*t*	1.176	0.715	0.375	0.002	*F*=0.937^※^ *P*=0.425	
	*P*	0.244	0.477	0.709	0.999		
CD8^+^CD28^-^	Docetaxol	14.3±6.1	12.5±10.0	15.4±8.1	15.8±7.0	0.783	0.471
	Pemetrexed	17.4±8.6	11.9±5.6^*^	14.3±8.1	13.5±8.3	2.626	0.057
	Sum	15.9±7.6	12.1±7.9	14.8±8.1	14.6±7.7	2.051	0.110
	*t*	1.029	0.036	0.545	0.590	*F*=1.093^※^ *P*=0.354	
	*P*	0.307	0.972	0.588	0.558		
^&^CT is abbreviation of chemotherapy; ^*^P < 0.05 in multiple comparisons against pro-CT; ^#^*t*’ test instead when *Levene's* test showed varience heterogeneity; ^※^*F* of interaction effect.							

### 2.4 一线化疗2周期后疾病进展（progressive disease, PD）的患者与部分缓解（partial response, PR）患者T淋巴细胞亚群变化的统计分析

将检测结果根据随访情况进行分层分析。49例患者经一线化疗2周期后，5例患者PD，14例患者PR，其余患者稳定（stable disease, SD）。将PD与PR患者化疗后第4、7-10及21天的外周血T淋巴细胞进行比较，结果显示：两组患者化疗后第10天CD3^+^均升高；PR组患者化疗后第4天及第7-10天CD3^+^CD4^+^细胞升高；化疗后第4天CD3^+^CD8^+^、CD8^+^CD28^-^细胞降低，差异有统计学意义（表 4）。

**4:** PD与PR患者T细胞亚群检测结果（Mean±SD, %） The T lymphocyte subset seperately in PD and PR group (Mean±SD, %)

T lymph-ocyte subset	Item	pro-CT	The 4^th^ day of CT	The 10^th^ day of CT	The 21^th^ day of CT	*F*	*P*
CD3^+^	PD	67.6±8.3	69.1±9.7	70.4±6.6^*^	67.4±8.3	3.114	0.067
	PR	65.7±12.0	67.4±14.4	67.9±14.0^*^	66.6±10.9	2.111^△^	0.142
	Sum	66.2±11.0	67.8±13.1	68.6±12.3	66.8±10.0	3.577^△^	0.034
	*t*	0.675	0.250	0.375	0.584	*F*=0.333^△※^ *P*=0.738	
	*P*	0.508	0.805	0.712	0.566		
CD3^+^CD4^+^	PD	41.6±7.6	44.1±8.2	42.0±6.4	41.3±6.5	0.161	0.920
	PR	36.4±12.2	43.6±10.0^*^	41.9±12.9^*^	37.1±6.5	4.546	0.008
	Sum	37.8±11.3	43.7±9.3	41.9±11.4	38.2±6.6	1.927	0.137
	*t*	1.139	0.088	0.004	2.034	*F*=0.628^※^ *P*=0.600	
	*P*	0.269	0.931	0.997	0.057		
CD3^+^CD8^+^	PD	24.1±8.7	26.7±9.8	25.6±4.4	24.7±8.9	0.115	0.949
	PR	27.2±7.6	20.6±6.7^*^	25.2±8.8	27.4±7.9	3.409^△^	0.047
	Sum	26.4±7.8	22.2±7.9	25.3±7.8	26.7±7.9	0.363	0.780
	*t*	0.647	1.565	0.099	1.038	*F*=1.500^※^ *P*=0.226	
	*P*	0.525	0.136	0.922	0.313		
CD4^+^/CD8^+^	PD	1.4±0.9	1.9±1.0	1.6±0.7	1.9±0.8	0.414	0.746
	PR	1.4±0.9	2.2±1.0	1.8±1.1	1.3±0.5	3.743	0.019
	Sum	1.4±0.9	2.1±1.0	1.7±1.0	1.4±0.6	1.650	0.189
	*t*	0.243	0.628	0.404	2.744	*F*=0.930^※^ *P*=0.433	
	*P*	0.811	0.539	0.691	0.013		
CD4^+^CD25^+^	PD	10.8±2.4	10.4±1.5	10.9±2.5	10.1±2.2	0.156	0.924
	PR	10.7±4.4	11.9±4.3	10.6±4.8	9.8±2.7	0.760	0.523
	Sum	10.7±3.9	11.5±3.8	10.7±4.3	9.9±2.5	0.334	0.801
	*t*	0.162	1.102^#^	0.132	0.458	*F*=0.231^※^ *P*=0.874	
	*P*	0.873	0.286	0.897	0.653		
CD8^+^CD28^+^	PD	12.7±5.6	14.9±6.2	18.0±3.4	12.8±3.0	2.491	0.110
	PR	15.1±4.4	13.5±3.8	13.9±4.8	15.8±4.1	1.171	0.333
	Sum	14.5±4.7	13.9±4.4	15.0±4.7	15.0±4.0	0.912	0.442
	*t*	1.313	0.613	1.777	1.599	*F*=3.079^※^ *P*=0.036	
	*P*	0.205	0.548	0.093	0.127		
CD8^+^CD28^-^	PD	13.4±6.5	13.1±6.0	11.0±3.0	11.8±6.5	0.211	0.887
	PR	17.7±10.0	10.2±4.7^*^	15.2±7.7	19.3±8.3	4.905	0.005
	Sum	15.0±4.0	10.9±5.1	14.1±7.0	17.3±8.4	1.312	0.281
	*t*	0.731	1.120	1.155	1.599	*F*=1.710^※^ *P*=0.177	
	*P*	0.474	0.278	0.264	0.040		
^△^*Mauchly's* test of sphericity *P* < 0.05，corrected by *Greenhouse-Geisser*; ^*^P < 0.05 in multiple comparisons against pro-CT; ^#^*t*’ test instead when *Levene's* test showed varience heterogeneity; PD: progressive disease; PR: partial response.							

## 3 讨论

机体免疫功能在抗肿瘤免疫应答中起着重要作用，近年来肺癌患者的免疫状态与患者的肿瘤临床病理学特征以及与预后的关系成为肿瘤研究焦点之一。化疗是晚期肺癌的主要治疗手段。探究化疗对免疫功能的影响和免疫功能与肿瘤治疗效果的关系，对于提高肿瘤综合治疗水平、加强对预后的认识有着重要的临床意义。

多西他赛与培美曲塞联合顺铂方案由于其安全性好、可耐受度高、疗效肯定及住院时间短等原因，是目前临床上晚期非小细胞肺癌尤其是肺腺癌一线治疗中最常采用的方案。本研究采用前瞻性试验设计，入组患者随机进入两种化疗方案组，总结试验结果，分析这两种化疗方案对机体免疫功能的影响，以期为化疗与免疫治疗的结合及提高临床疗效提供依据。T淋巴细胞是细胞免疫的效应细胞，在肿瘤免疫中起中心调控作用。检测患者T淋巴细胞表达水平，可以辅助动态分析抗肿瘤药物治疗过程中机体细胞免疫功能的变化。

首先使用流式细胞检测法分析来自于49例晚期肺腺癌患者的外周血的CD3^+^、CD4^+^、CD8^+^和CD8^+^CD28^+^、CD8^+^CD28^-^、CD4^+^CD25^+^T细胞占外周血淋巴细胞的比例，并与正常人进行比较。CD3^+^CD4^+^T为辅助T淋巴细胞，CD3^+^CD8^+^T为抑制T淋巴细胞。正常情况下，CD3^+^CD8^+^和CD3^+^CD4^+^之间保持着动态平衡，CD4^+^/CD8^+^下降表示患者处于免疫抑制状态。CD8^+^T细胞根据CD28的表达与否分为抑制性T细胞（Ts，人类表型为CD8^+^CD28^-^）和杀伤性T细胞（Tc，人类表型为CD8^+^CD28^+^）。CD8^+^CD28^-^T细胞存在于肿瘤组织中，是一群终末分化的细胞，被认为具有抑制T、B细胞活性和介导细胞毒活性的功能^[5]^。CD4^+^CD25^+^T（Treg）细胞是一类具有负免疫调节作用的T淋巴细胞亚群。Treg细胞可以抑制识别自身肿瘤细胞的效应细胞的发育和活化，在介导机体肿瘤免疫耐受中起重要作用^[6]^。近年来研究^[7]^表明，许多恶性肿瘤患者外周血和肿瘤局部微环境中的Treg细胞比例都明显增高，且数量还与患者肿瘤进展程度和预后呈负相关。控制Treg的数量和功能有可能是肿瘤免疫治疗的新策略^[8]^。

本实验结果显示：肺癌患者外周血CD3^+^、CD3^+^CD4^+^细胞比例明显低于健康组，*P* < 0.05。CD4^+^CD25^+^（Treg）细胞比例明显高于健康组，*P* < 0.01。CD4^+^/CD8^+^、CD8^+^CD28^+^、CD8^+^CD28^-^与正常人相比无明显差异。恶性肿瘤患者CD4^+^T细胞亚群的改变情况报道不一，但是多数研究显示CD4^+^T细胞亚群在恶性肿瘤患者中是表现为减少的。Treg细胞在多种实体瘤组织及患者外周血中均高于正常对照。说明肺癌患者细胞免疫功能处于抑制状态，符合肿瘤的免疫逃避机制。

近年的研究发现各种非遗传因素（如化疗药物）导致的淋巴细胞减少可诱发机体免疫系统的自稳性增生，从而使体内的T细胞数目保持在一定的水平。淋巴细胞自稳性增生可使肿瘤抗原特异性T淋巴细胞克隆在“广阔”的空间内扩增，增强抗肿瘤的免疫应答^[9]^。本实验结果显示，肺癌患者在化疗后CD3^+^升高，差异有统计学意义。CD3^+^CD4^+^细胞比例及CD4^+^/CD8^+^明显升高，CD8^+^CD28^-^明显降低，结果具统计学意义。后各细胞比例逐渐恢复，至第21天与化疗前基本相同。提示化疗不仅没有削弱机体免疫功能，且能诱使机体朝正向免疫应答发展。

本实验还分析了不同化疗药物对患者免疫功能影响的差别。已有许多报道^[4, 10-15]^证实环磷酰胺、多西他赛、氟达拉滨等化疗药物及索拉非尼、抗CD25单抗类靶向药物等对Treg起选择性抑制作用。而其它化疗药物对Treg细胞的影响尚未十分明确。为观察不同化疗药物对免疫功能的影响，本实验组患者随机进入多西他赛组及培美曲赛组，两组患者均为Ⅲb期-Ⅳ期肺腺癌患者，性别、年龄等因素均无明显差异。实验结果表明，化疗后短期内两组患者的效应性T细胞CD3^+^CD4^+^均出现明显升高，且培美曲塞组更为明显，持续时间更长，且伴有CD3^+^CD8^+^及CD8^+^CD28^-^等抑制性细胞成分的明显减少。结果提示与多西他塞组比较，培美曲塞似乎对化疗后短期内免疫功能的改善有更明显的作用。根据既往文献提示，我们预测多西他赛组可能出现Treg细胞的特异性抑制。然而我们着重观察了化疗前后两组患者的Treg细胞比例，并未观察到明显变化，甚至在化疗后第4天Treg细胞数量还有所升高，但差异无统计学意义。分析原因，可能与化疗合并使用小剂量糖皮质激素进行预处理有关。为了防止皮疹、水钠潴留及过敏反应等，临床实际应用培美曲塞及多西他赛时，需在化疗前一天、化疗当天及化疗后一天口服小剂量地塞米松。实验^[14]^表明，糖皮质激素可明显提升外周血Treg细胞数量。激素可能既可抑制效应性T细胞，又可上调调节性T细胞的比例，也可能增强了调节性T细胞的免疫抑制功能。化疗药物虽然可能选择性地清除CD4^+^CD25^+^FoXP3^+^Treg细胞，但其作用不敌糖皮质激素的影响。另外，化疗药物不同的给药方式以及研究对象不同的病理类型、疾病分期对免疫功能产生的影响也不同。因此在本实验中，化疗短期内CD4^+^CD25^+^总数量表现为上升的结果。而化疗7 d-10 d后，化疗对机体的短期影响结束，Treg细胞含量可能升高。因此结合临床实际，本研究在晚期肺腺癌联合化疗对Treg细胞的影响观察中未复制出与文献报道相似的结果。同样，虽然文献报道多西他赛在动物实验中可选择性抑制Treg细胞，但将多西他赛与培美曲塞进行分层分析，并未得出特异性结果。培美曲塞组在化疗后第4天CD3^+^细胞比例升高更明显，提示似乎培美曲塞对化疗后短期内免疫功能的改善有更明显的作用。

本实验初步探讨了T细胞亚群与近期疗效的关系。结果显示，PR组患者化疗后第4天及第7-10天CD3^+^CD4^+^细胞升高，化疗后第4天CD3^+^CD8^+^、CD8^+^CD28^-^细胞降低（*P* < 0.05）。其余细胞成分未观察到明显变化。似乎提示化疗后体内免疫环境有所改善。但病例数太少，统计结果可能存在偏差。因此该结果是否提示化疗后对体内免疫格局的变化与预后有明确关系，尚需要更大规模的临床试验进一步证实。

以上结果提示化疗对机体免疫微环境具有一定的优化作用，能活化抗肿瘤免疫应答，增强机体的抗肿瘤反应。临床实际应用中可能影响患者免疫功能的原因很多，诱发抗肿瘤免疫活化的因素还不完全了解。而化疗药物对机体免疫细胞的作用机制以及化疗药物在第几个周期后发挥明显作用更是知之甚少。免疫治疗在通往临床应用的途中，还有许多关键问题有待解决，包括合并化疗药的给药方式、免疫治疗的切入时机等。而糖皮质激素在肿瘤患者体内引起的免疫功能改变是否影响到肿瘤的发生发展以及化疗效果，也需要更多实验的验证。
